# Comparative Analysis of Exonic SSR‐Containing Genes in Three Diving Duck Species Reveals Shared Functional Themes

**DOI:** 10.1002/ece3.74632

**Published:** 2026-11-01

**Authors:** Jie Huang, Bo Yang, Jiayi Qiao, Huaming Zhong, Chengzhong Yang

**Affiliations:** ^1^ College of Biology and Food Shangqiu Normal University Shangqiu Henan China; ^2^ China Conservation and Research Centre for the Giant Panda Chengdu Sichuan China; ^3^ Animal Biology Key Laboratory of Chongqing Education Commission of China, Chongqing Key Laboratory of Conservation and Utilization of Freshwater Fishes, College of Life Sciences Chongqing Normal University Chongqing China

**Keywords:** avian genomics, comparative genomics, diving ducks, exonic SSRs, gene ontology, KEGG, simple sequence repeats

## Abstract

Simple sequence repeats (SSRs) are ubiquitous components of eukaryotic genomes that contribute to genome evolution, genetic variation, and gene regulation. Although genome‐wide SSR landscapes have been extensively characterized across vertebrates, the cross‐species distribution and functional associations of exonic SSR‐containing genes remain poorly understood, particularly in non‐model birds. To address this knowledge gap, we performed a comparative analysis of chromosome‐level genome assemblies from three diving duck species ( *Aythya ferina* , *Aythya marila* , and *Netta rufina* ) to investigate genome‐wide SSR patterns and the cross‐species sharing and functional characteristics of exonic SSR‐containing genes. Comparative analyses of genome‐wide SSRs revealed pronounced interspecific variation in SSR abundance and repeat composition, whereas exonic SSR abundance showed more similar values after normalization by total annotated exon sequence length. Cross‐species comparison identified 4336 exonic SSR‐containing genes shared among all three species, greatly outnumbering the species‐specific gene sets. Functional enrichment analyses identified four broad functional themes associated with these shared genes: (i) gene transcription and epigenetic regulation, (ii) protein homeostasis and the ubiquitin–proteasome system, (iii) development, differentiation and morphogenesis, and (iv) stress response and cellular maintenance. The most significantly enriched categories were associated with transcriptional regulation, chromatin remodeling, RNA processing, and epigenetic regulation, whereas autophagy‐ and other cellular‐maintenance pathways indicated additional associations with stress‐response processes. These functional associations should be interpreted cautiously because they may be influenced by cross‐species annotation limitations and gene‐architecture differences. Collectively, our results reveal distinct patterns for genome‐wide and exonic SSRs distribution among the three diving duck species and identify shared functional associations of genes containing exonic SSRs. These findings provide a comparative genomic framework for future investigations of exonic SSRs in waterfowl and other non‐model vertebrates.

## Graphical abstract

Comparative analysis of chromosome‐level genomes from three diving duck species revealed pronounced interspecific variation in genome‐wide SSR abundance and repeat composition, while identifying 4336 exonic SSR‐containing genes shared among all three species. Functional enrichment analyses of these shared genes highlighted four broad functional themes related to transcription and epigenetic regulation, protein homeostasis, development, and stress response, although these associations should be interpreted cautiously in light of annotation limitations and potential gene‐architecture bias. This study provides a comparative genomic framework for future investigations of exonic SSRs in waterfowl and other non‐model vertebrates.

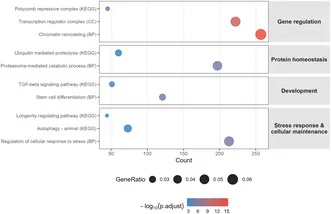

## 1 Introduction

Simple sequence repeats (SSRs), also known as microsatellites, are tandemly repeated DNA motifs consisting of one to six nucleotides that are widely distributed throughout eukaryotic genomes (Tóth et al. 2000). Owing to their high mutation rates, codominant inheritance, and extensive genomic distribution, SSRs have long been used as powerful molecular markers for population genetics, linkage mapping, parentage analysis, and conservation biology (Ellegren 2004; Kulibaba et al. 2025). Increasing evidence, however, indicates that microsatellites are not merely neutral genetic markers but also represent important components of genome architecture that may influence genome evolution, gene regulation, and phenotypic diversification (Li et al. 2004; Gemayel et al. 2010). The rapid accumulation of chromosome‐level avian genomes, together with large‐scale initiatives such as the Duck 1000 Genomes Project, has greatly facilitated comparative genomic analyses of repetitive sequences and genome evolution (Fan et al. 2024). This genomic resource is particularly valuable for investigating repetitive DNA in birds, where microsatellites and other repetitive elements contribute substantially to genome organization and chromosome evolution (Kretschmer et al. 2018; Feng et al. 2020). Recent microsatellite mapping across Anseriformes further demonstrates that repetitive sequences contribute to chromosomal diversification during waterfowl evolution (Campos et al. 2025).

Comparative genomics has substantially advanced our understanding of avian genome evolution over the past decade. Dense phylogenomic sampling across birds has revealed that, despite the overall structural conservation of avian genomes, repetitive sequences remain highly dynamic and contribute to lineage‐specific genome evolution (Feng et al. 2020). More recently, chromosome‐level comparative analyses demonstrated considerable variation in SSR abundance, motif composition, and chromosomal distribution among bird species, indicating that microsatellite evolution differs markedly across avian lineages (Feng et al. 2023). Similar patterns have been reported in other organisms, where SSR abundance and composition vary among genomic regions, suggesting that local genomic context exerts an important influence on microsatellite evolution (Zhao et al. 2023). Recent studies have begun to characterize genome‐wide microsatellites in ducks, facilitating marker development and comparative genomic analyses, although these investigations have focused primarily on genome‐wide SSR identification rather than exonic SSRs (Safaa et al. 2023; Sivalingam et al. 2025). Despite these advances, most previous studies have primarily described genome‐wide SSR landscapes or developed SSR markers for genetic applications (Guo et al. 2025; Kulibaba et al. 2025). Consequently, the functional significance and evolutionary dynamics of SSRs located within exon regions remain poorly understood.

Exonic SSRs represent a particularly important subset of microsatellites because sequence variation within exonic regions may affect gene function in a context‐dependent manner. The potential consequences depend on the genomic position of the repeat within an exon, including whether it occurs in a coding or untranslated exon. Compared with SSRs located in non‐coding regions, such as intergenic and intronic regions, exonic SSRs may be subject to different evolutionary constraints because changes in repeat length can affect the sequence or regulatory properties of the host gene. Previous studies have suggested that SSRs located in coding or other functionally constrained genomic regions may influence transcription, alternative splicing, protein properties, or other aspects of gene regulation (Li et al. 2004; Gemayel et al. 2010; Kashi and King 2006). Comparative analyses have also indicated that SSRs exhibit distinct evolutionary characteristics across genomic compartments (Zhao et al. 2023). However, whether exonic SSRs are preferentially distributed among genes with specific biological functions remains poorly understood, particularly in avian genomes.

Diving ducks (tribe Aythyini) provide an attractive system for investigating these questions. Members of this lineage have evolved a series of specialized physiological, morphological, and metabolic adaptations that enable prolonged underwater foraging, making them valuable models for studying the genomic basis of ecological adaptation. The availability of chromosome‐level genome assemblies for multiple diving duck species has substantially expanded genomic resources for investigating the evolutionary basis of these adaptations (Brown et al. 2022; Xia et al. 2024, 2026; Ding et al. 2025). More broadly, comparative genomic studies of aquatic birds, including penguins, have demonstrated that repeated transitions to aquatic lifestyles are accompanied by convergent genomic adaptations affecting metabolism, oxygen utilization, and environmental responses (Cole et al. 2022). Despite these advances, microsatellite evolution has received relatively little attention in comparative studies of diving waterfowl, and no comparative genomic study has systematically investigated the cross‐species distribution and functional associations of exonic SSR‐containing genes across multiple diving duck species.

In this study, we performed the first comparative genomic analysis of genome‐wide and exonic SSRs in three representative diving duck species ( *Aythya ferina* , *A. marila* , and *Netta rufina* ). Specifically, we (i) characterized genome‐wide SSR landscapes, (ii) identified exonic SSR‐containing genes, (iii) examined the extent of gene‐set sharing among the three species, and (iv) investigated the functional associations of shared exonic SSR‐containing genes using GO and KEGG enrichment analyses. We selected *A. ferina* , *A. marila* , and *N. rufina* because chromosome‐level genome assemblies and corresponding genome annotations were available for all three species, enabling a comparable analysis of genome‐wide and exonic SSR landscapes across representatives of the *Aythya* and *Netta* lineages. By integrating comparative genomics with functional enrichment analyses, this study aims to characterize the comparative distribution and functional associations of exonic SSRs and to provide a genomic framework for future investigations of exonic SSR evolution in waterfowl and other avian taxa.

## 2 Methods

### 2.1 Genome Assemblies and Annotation Data

Chromosome‐level genome assemblies of three diving duck species, *Aythya ferina* (GCF_964211825.1), *Aythya marila* (GCF_965140915.1), and *Netta rufina* (GCF_964035555.1), were downloaded from the NCBI GenBank database. The corresponding genome annotation (GFF3) files were retrieved simultaneously for gene annotation and exon coordinate extraction. The three assemblies exhibited high completeness, with scaffold N50 values of 79.8, 77.0, and 77.3 Mb and BUSCO completeness scores of 98.0%, 97.9%, and 98.1%, respectively (Supplementary Table S1).

### 2.2 Genome‐Wide SSR Identification

Genome‐wide simple sequence repeats (SSRs) were identified using Krait v2.0.6 (Du et al. 2018). Only perfect (uninterrupted) SSRs were considered in this study. Minimum repeat thresholds were set to ≥ 12 repeat units for mono‐nucleotide motifs, ≥ 7 for di‐nucleotide motifs, ≥ 5 for tri‐nucleotide motifs, and ≥ 4 for tetra‐, penta‐, and hexa‐nucleotide motifs, following previously established criteria (Huang et al. 2015, 2016).

For each genome, the total number of SSR loci, SSR abundance (loci Mb^−1^), repeat‐type composition (mono‐ to hexa‐nucleotide), and motif distribution were calculated. The same analytical procedure was subsequently applied to SSRs overlapping exon regions, with exonic SSR abundance calculated as the number of unique exonic SSR loci per Mb of total exon sequence.

### 2.3 Identification of Exonic SSR‐Containing Genes

Exon coordinates were extracted from the corresponding GFF3 annotation files by selecting features annotated as exon. SSR loci overlapping annotated exon intervals were identified using BEDTools. An SSR locus was considered exonic when its genomic interval overlapped an annotated exon interval by at least 1 bp. An exonic SSR‐containing gene was defined as a gene containing at least one exonic SSR locus. Transcript‐to‐gene relationships recorded in the GFF3 annotations were used to associate overlapping exon features, and consequently the corresponding exonic SSR loci, with gene identifiers.

Gene symbols were extracted from the genome annotations and standardized across the three species. Standardized gene symbols were used for gene‐level cross‐species comparison, whereas Entrez Gene IDs were used for downstream functional enrichment analyses. The gene‐level comparison was based on standardized gene‐symbol correspondence and was not intended to constitute a dedicated orthology‐inference analysis. Human Entrez Gene IDs were assigned through symbol‐based matching against the **org.Hs.eg.db** annotation database because a dedicated Anatidae annotation package was not available. This procedure was used for identifier harmonization and functional annotation rather than for establishing one‐to‐one orthologous relationships. Genes without valid standardized symbols or successfully assigned Entrez Gene IDs were excluded from the corresponding downstream analyses.

### 2.4 Construction of the Common Background Gene Universe

To enable direct functional comparisons among the three species, a common background gene universe was established. For each species, all successfully annotated genes with valid Entrez Gene IDs were first compiled as the initial background gene set. The intersection of the three species‐specific background sets yielded a common reference gene universe comprising 12,680 Entrez Gene IDs. This intersection‐based background was selected to restrict the reference population to genes represented across all three species, thereby improving comparability and reducing differences caused by species‐specific annotation coverage. It excludes genes represented in only one or two species and therefore does not represent the complete gene complement of each species.

### 2.5 Construction of Independent Background Sets for Gene‐Architecture Analysis

To construct independent background sets for gene‐architecture analysis, the common background Entrez ID set was first converted to gene symbols. Duplicate symbols were collapsed before comparison with the shared foreground set. The 4336 shared exonic SSR‐containing gene symbols were then excluded from the deduplicated background symbol set based on actual set membership rather than by simple numerical subtraction. The resulting background set contained 8348 unique genes in each species. These genes were subsequently mapped to the corresponding species‐specific locus tags to retrieve annotated exon lengths and gene genomic spans from the GFF3 annotations.

### 2.6 Identification of Shared and Species‐Specific Exonic SSR‐Containing Genes

Shared and species‐specific exonic SSR‐containing genes were identified by comparing the three species‐specific gene sets. Genes detected in all three species were classified as shared genes, whereas genes unique to a single species were defined as species‐specific genes. Venn diagrams were generated using the VennDiagram package (v1.8.2) (Chen and Boutros 2011).

### 2.7 Gene Ontology and KEGG Enrichment Analyses

Functional enrichment analyses were performed using the clusterProfiler package (v4.18.4) (Yu et al. 2012). Gene symbols of shared and species‐specific exonic SSR‐containing genes were converted to Entrez Gene IDs using the org.Hs.eg.db annotation database because a dedicated Anatidae annotation package was not available. For the shared‐gene analysis, the 4336 shared exonic SSR‐containing genes were used as the foreground set, and the common background gene universe of 12,680 Entrez Gene IDs was used as the reference population. The nominal foreground set was defined as the 4336 shared genes; the effective foreground sizes used by individual enrichment functions were determined by the genes with valid annotations in the corresponding database. Gene Ontology (GO) enrichment analyses were conducted separately for the Biological Process (BP), Cellular Component (CC), and Molecular Function (MF) using the enrichGO function with keyType = “ENTREZID”, pAdjustMethod = “BH”, pvalueCutoff = 0.05, and qvalueCutoff = 0.05. KEGG pathway enrichment was performed using the enrichKEGG function with organism = “hsa” and the same significance threshold and multiple‐testing correction. Because GO and KEGG provide different annotation spaces, the numbers of genes represented in the effective annotation sets differed between analyses. Specifically, 4051 of the shared genes were represented in the GO annotation space, whereas 2010 were represented in the KEGG annotation space; the corresponding effective background sets comprised 11,722 and 5885 genes, respectively. Species‐specific enrichment analyses were performed separately for each species using its corresponding species‐specific background gene universe. Significant GO terms and KEGG pathways were identified at an adjusted *p* value < 0.05.

For biological interpretation, significantly enriched GO terms and KEGG pathways from the shared‐gene analysis were manually grouped into four broad functional themes according to their biological relevance and conceptual similarity. These groupings were used for presentation rather than representing an independent statistical clustering or network analysis. The order of the four themes was used for presentation and did not represent a formal ranking of biological importance or statistical significance.

### 2.8 Assessment of Gene‐Architecture Bias

To evaluate whether gene architecture could influence the occurrence or detection of exonic SSRs, we compared summed annotated exon length and gene genomic span between the shared exonic SSR‐containing genes and independent background genes in each species. The common background set initially contained 12,680 Entrez IDs, corresponding to 12,678 unique gene symbols after symbol‐level deduplication. Of these, 4330 symbols were also present in the shared foreground set. After excluding these overlapping symbols, the final independent background set contained 8348 genes in each species.

Summed annotated exon length was calculated by summing the lengths of all exon features assigned to the same gene according to the species‐specific GFF3 annotation. This measure represents summed annotated exon length rather than non‐redundant genomic exon span and may include overlapping regions associated with alternative transcripts. Gene genomic span was defined as the genomic span of the annotated gene feature, calculated as gene end − gene start + 1. The resulting gene sets were mapped to species‐specific locus tags to retrieve the corresponding length measurements.

Differences in length distributions between shared and background gene sets were assessed using two‐sided Mann–Whitney *U* tests in R. This analysis was designed to evaluate a potential source of gene‐architecture bias and was not intended to statistically correct the subsequent functional enrichment analyses.

### 2.9 Statistical Analysis and Visualization

All statistical analyses and graphical visualizations were performed in R (v4.5.3). Figures were generated using ggplot2 (v4.0.3) (Wickham 2016) and assembled using patchwork (v1.3.2) (Pedersen 2025). Bar plots were used to compare genome‐wide and exonic SSR abundance, repeat‐type composition, and motif distribution among species. Bubble plots were used to visualize representative GO terms and KEGG pathways. The horizontal axis represents Count, defined as the number of input genes assigned to each term, point size represents GeneRatio, calculated as Count divided by the total number of genes successfully analyzed in the corresponding enrichment dataset, and point color represents enrichment significance [−log_10_(adjusted *p* value)].

## 3 Results

### 3.1 Genome‐Wide and Exonic Distribution of SSRs in Three Diving Duck Genomes

To investigate the genomic landscape of simple sequence repeats (SSRs) in diving ducks, perfect SSRs were identified from the chromosome‐level genome assemblies of *Aythya ferina* , *Aythya marila* , and *Netta rufina* . The three genomes were highly comparable in size, ranging from 1166 Mb in *N. rufina* to 1252 Mb in *A. ferina* , with GC contents of approximately 42% (Supplementary Table S1). Despite these similarities, substantial variation was observed in genome‐wide SSR abundance among species. A total of 884,701, 907,250, and 605,018 SSR loci were detected in *A. ferina* , *A. marila* , and *N. rufina* , respectively, corresponding to relative abundances of 706.46, 740.90, and 518.46 loci per Mb (Figure 1A,B; Table 1). Compared with the two *Aythya* species, *N. rufina* possessed markedly fewer genome‐wide SSRs while exhibiting a comparable genome size.

**FIGURE 1:**
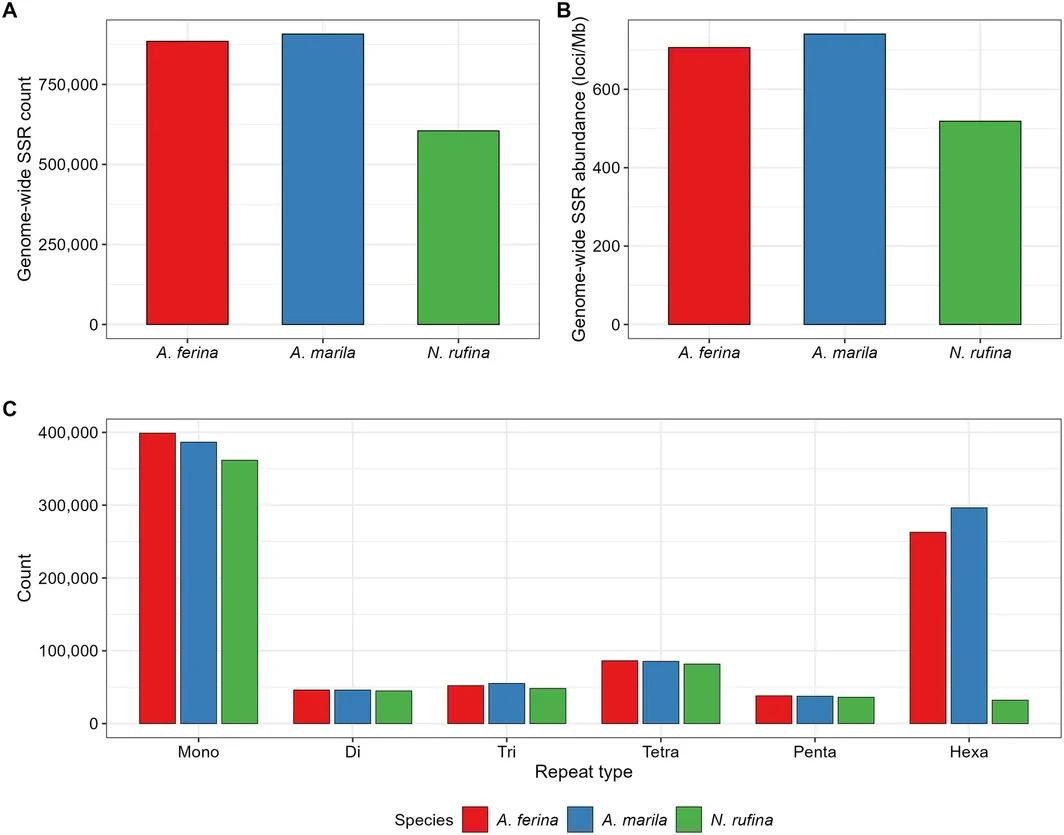
Genome‐wide SSR characteristics of three diving duck species. (A) Total number of SSR loci identified in each genome. (B) Relative abundance of SSRs (loci per Mb). (C) Distribution of SSR loci by repeat type (mono‐ to hexa‐nucleotide). Bars represent counts for each repeat type, with species shown in different colors: *A. ferina* (red), *A. marila* (blue), and *N. rufina* (green). Mono‐, di‐, tri‐, tetra‐, penta‐, and hexa‐nucleotide repeats are abbreviated as Mono, Di, Tri, Tetra, Penta, and Hexa, respectively.

**TABLE 1:** Genome‐wide and exonic SSR statistics of three diving duck species.

Species	Genome size (Mb)	Genome‐wide SSR loci	Genome‐wide SSR abundance (loci/Mb)	Exonic SSR loci	Exonic SSR abundance (loci/Mb exon)
*Aythya ferina*	1252	884,701	706.46	26,550	114
*Aythya marila*	1224	907,250	740.9	20,131	140
*Netta rufina*	1166	605,018	518.46	13,064	138

*Note:* Genome‐wide SSR abundance was calculated as the number of genome‐wide SSR loci per megabase of genome sequence, whereas exonic SSR abundance was calculated as the number of SSR loci overlapping exon regions per megabase of total exon sequence.

The composition of genome‐wide SSRs also differed among species (Figure 1C). Mono‐nucleotide repeats represented one of the most abundant repeat classes in all three genomes, with 398,895 loci in *A. ferina* , 386,550 in *A. marila* , and 361,685 in *N. rufina* . Hexa‐nucleotide repeats were similarly abundant in the two *Aythya* species, accounting for 262,897 and 296,412 loci, respectively, whereas only 32,206 hexa‐nucleotide repeats were identified in *N. rufina* . In contrast, the numbers of di‐, tri‐, tetra‐, and penta‐nucleotide repeats varied only slightly among species, ranging from 44,902–46,171, 48,341–55,145, 81,745–86,337, and 36,139–38,169 loci, respectively. These results indicate that the principal difference in genome‐wide SSR composition among the three species was attributable to variation in hexa‐nucleotide repeats.

Because SSRs located within exon regions were of particular interest in this study, exonic SSRs were analyzed separately. The numbers of exonic SSRs were 26,550 in *A. ferina* , 20,131 in *A. marila* , and 13,064 in *N. rufina* (Figure 2A; Table 1). When normalized by total exon sequence length, the exonic SSR abundances were 114.0, 140.0, and 138.0 loci per Mb of exon sequence in *A. ferina* , *A. marila* , and *N. rufina* , respectively (Figure 2B; Table 1). Although *N. rufina* exhibited the lowest absolute number of exonic SSR loci, its normalized abundance was comparable to that of *A. marila* and exceeded that of *A. ferina* .

**FIGURE 2:**
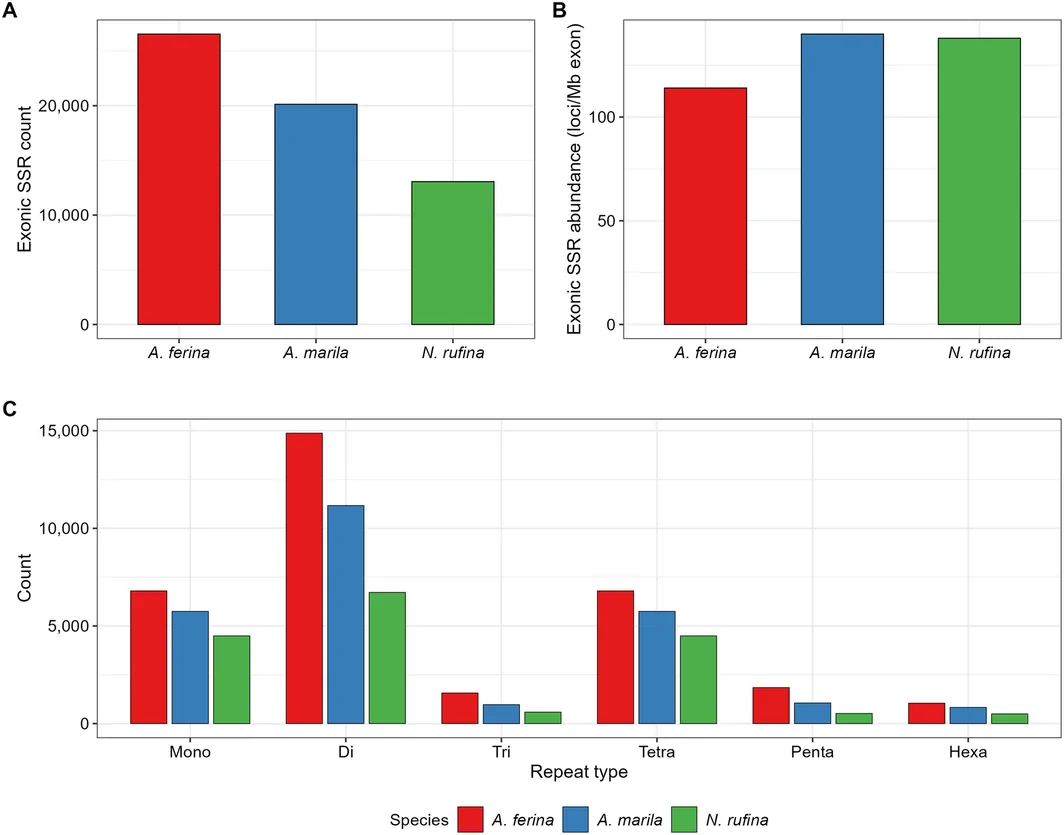
Exonic SSR characteristics of three diving duck species. (A) Number of exonic SSR loci. (B) Exonic SSR abundance (loci per Mb of exon sequence). (C) Distribution of exonic SSR loci by repeat type (mono‐ to hexa‐nucleotide). Bars represent counts for each repeat type, with species shown in different colors: *A. ferina* (red), *A. marila* (blue), and *N. rufina* (green). Mono‐, di‐, tri‐, tetra‐, penta‐, and hexa‐nucleotide repeats are abbreviated as Mono, Di, Tri, Tetra, Penta, and Hexa, respectively.

The repeat composition of exonic SSRs differed markedly from that observed across the whole genome (Figure 2C). Di‐nucleotide repeats were the predominant class in all three species, accounting for 14,875 loci in *A. ferina* , 11,169 in *A. marila* , and 6718 in *N. rufina* , followed by mono‐nucleotide repeats with 6795, 5746, and 4493 loci, respectively. Tri‐, tetra‐, penta‐, and hexa‐nucleotide repeats each represented substantially smaller proportions, with *N. rufina* consistently exhibiting lower counts than the two *Aythya* species. Overall, the predominance of di‐nucleotide repeats in exonic regions contrasted with the genome‐wide enrichment of mono‐ and hexa‐nucleotide repeats, demonstrating distinct SSR composition patterns between exonic and whole‐genome sequences.

### 3.2 Shared and Species‐Specific Exonic SSR‐Containing Genes

To identify exonic SSR‐containing genes shared among the three diving duck species, we compared the species‐specific gene sets after gene‐symbol standardization. For each species, genes with valid Entrez Gene IDs were also compiled to construct a common background gene universe. The intersection of the three background sets yielded 12,680 shared Entrez IDs, representing 94.18%, 96.03%, and 96.98% of the mapped genes in *A. ferina* , *A. marila* , and *N. rufina* , respectively (Table 2). This high degree of overlap provided a consistent reference set for subsequent comparative functional analyses.

**TABLE 2:** Gene annotation and identifier mapping statistics for exonic SSR‐containing genes.

Item	*Aythya ferina*	*Aythya marila*	*Netta rufina*
Genome annotation			
Genes annotated in GFF3	25,350	18,009	16,443
Genes with Valid gene symbols	13,482	13,222	13,093
Genes mapped to Entrez IDs	13,464	13,204	13,075
Exonic SSR‐containing genes			
Exonic SSR‐containing genes	9300	8336	6055
Exonic SSR‐containing gene with valid symbols	7908	7313	5533
Exonic SSR‐containing genes mapped to Entrez IDs	7889	7301	5526

*Note:* Exonic SSR‐containing genes were defined as genes containing at least one SSR locus overlapping an annotated exon feature. Gene symbols were standardized and used for cross‐species comparison, whereas Entrez Gene IDs were used for downstream functional enrichment analyses. The numbers reported for genes with valid symbols and genes mapped to Entrez IDs represent successive identifier‐filtering steps and are not independent gene categories.

A total of 4336 exonic SSR‐containing genes were shared among all three species based on standardized gene symbols (Figure 3; Table 2). In contrast, species‐specific exonic SSR‐containing genes were relatively limited, numbering 1405 in *A. ferina* , 772 in *A. marila* , and 355 in *N. rufina* . Thus, the three species shared a substantial common component of exonic SSR‐containing genes, while each species also possessed a distinct subset of genes not shared by the other two species.

**FIGURE 3:**
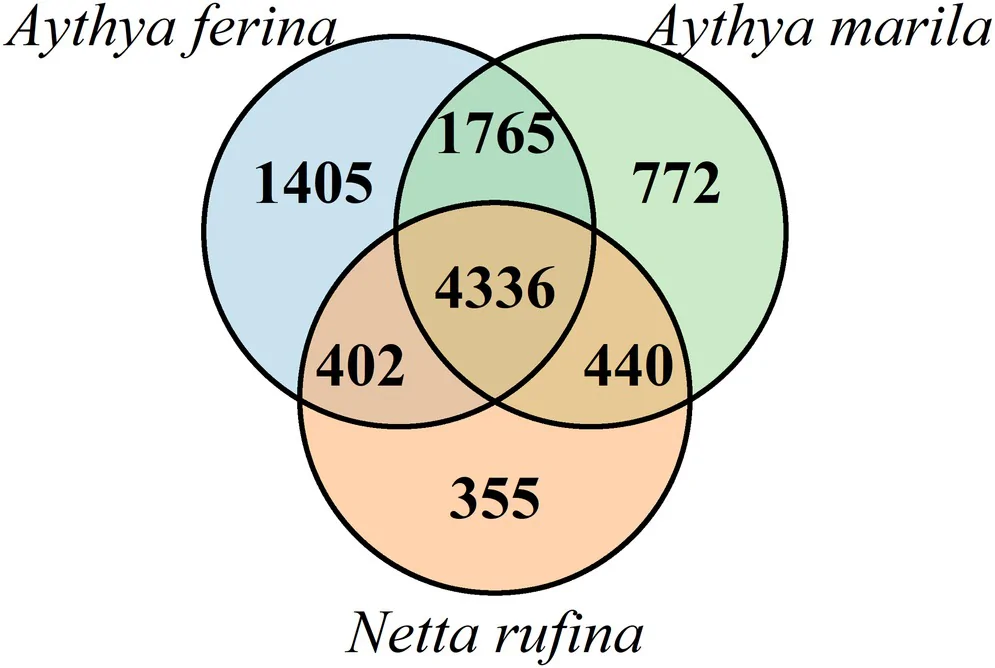
Shared and species‐specific exonic SSR‐containing genes among three diving duck species. The numbers in each region indicate the counts of genes containing at least one exonic SSR locus based on standardized gene symbols. A total of 4336 genes were shared among *A. ferina* , *A. marila* , and *N. rufina* , whereas species‐specific exonic SSR‐containing genes numbered 1405, 772, and 355 in *A. ferina*, *A. marila* , and *N. rufina* , respectively. The Venn diagram illustrates the extent of overlap and species‐specific components of exonic SSR‐containing gene sets.

In *A. ferina* , 9300 genes containing at least one exonic SSR were identified, of which 7908 had valid standardized gene symbols and 7889 could be mapped to Entrez IDs (Table 2). In *A. marila* , 8336 exonic SSR‐containing genes were identified, including 7313 genes with valid standardized symbols and 7301 genes with mapped Entrez IDs. In *N. rufina* , the corresponding numbers were 6055, 5533, and 5526, respectively. These reductions reflect the identifier‐standardization and Entrez‐mapping steps used to obtain gene sets compatible with the downstream comparative and enrichment analyses.

### 3.3 Functional Enrichment of Shared Exonic SSR‐Containing Genes

To investigate the biological functional characteristics of exonic SSR‐containing genes shared among the three diving duck species, GO and KEGG enrichment analyses were performed using the common background gene universe. The shared set comprised 4336 exonic SSR‐containing genes based on standardized gene symbols. After conversion to Entrez Gene IDs, 4051 shared genes were represented in the GO annotation space and 2010 in the KEGG annotation space. The corresponding effective background sets comprised 11,722 genes for GO and 5885 genes for KEGG, respectively. This difference reflects the distinct annotation coverage of GO and KEGG rather than additional biological filtering of the shared gene set. The significantly enriched GO terms and KEGG pathways were organized into four broad functional themes according to their biological relevance: (1) Gene transcription and epigenetic regulation, (2) Protein homeostasis and ubiquitin–proteasome system, (3) Development, differentiation and morphogenesis, and (4) Stress response and cellular maintenance (Figure 4; Supplementary Tables S2 and S3).

**FIGURE 4:**
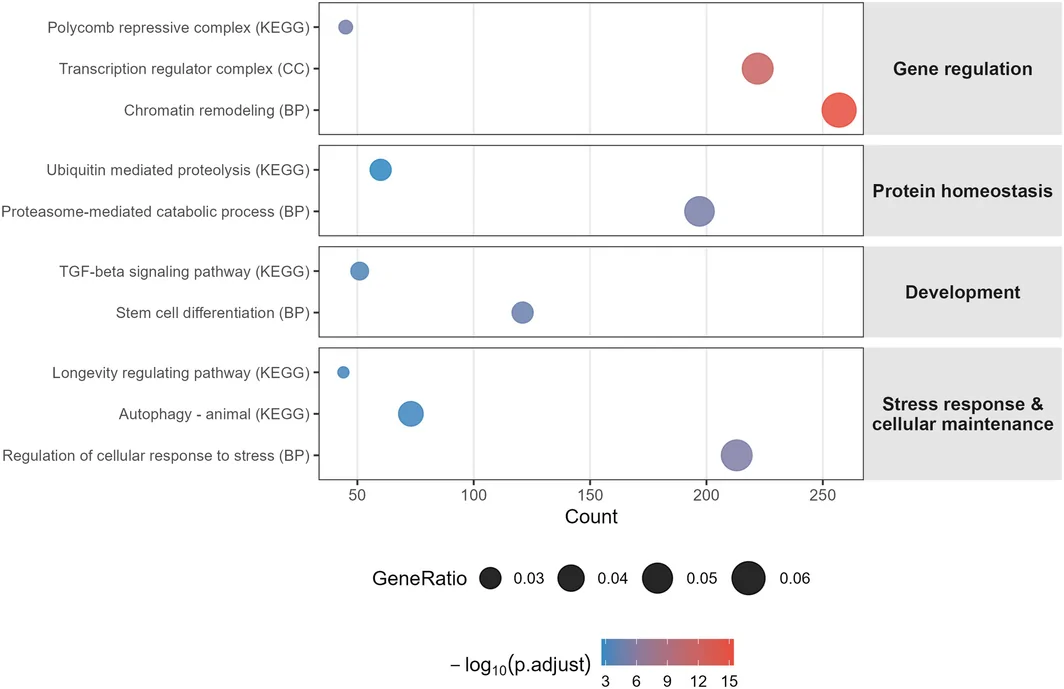
Functional enrichment patterns of the shared exonic SSR‐containing genes across the three diving duck species. The selected significantly enriched Gene Ontology (GO) and Kyoto Encyclopedia of Genes and Genomes (KEGG) terms are grouped into four broad functional themes: gene regulation, protein homeostasis, development, and stress and cellular maintenance. Each point represents one GO or KEGG term. The horizontal position indicates Count, defined as the number of input genes assigned to the corresponding term. Point size represents GeneRatio, calculated as Count divided by the total number of genes successfully analyzed in the corresponding enrichment dataset. Point color represents −log_10_(adjusted *p*‐value), with warmer colors indicating smaller adjusted *p* values. Terms are labeled with their ontology origin: BP, biological process; CC, cellular component; KEGG, Kyoto Encyclopedia of Genes and Genomes. The grouping of terms into functional themes was based on conceptual biological similarity and was used for presentation purposes.

The transcription and epigenetic regulation theme contained the greatest number of selected enriched terms, including 11 GO terms and two KEGG pathways. Among biological processes, chromatin remodeling (GO:0006338; 257 genes; adjusted *p* = 3.70 × 10^−16^), regulation of mRNA metabolic process (GO:1903311; 135 genes; adjusted *p* = 2.49 × 10^−5^), alternative mRNA splicing via spliceosome (GO:0000380; 46 genes; adjusted *p* = 2.47 × 10^−4^), and epigenetic regulation of gene expression (GO:0040029; 103 genes; adjusted *p* = 2.56 × 10^−4^) were significantly enriched. At the cellular component level, transcription regulator complex (GO:0005667; 222 genes; adjusted *p* = 1.84 × 10^−12^) and RNA polymerase II transcription regulator complex (GO:0090575; 102 genes; adjusted *p* = 9.63 × 10^−5^) showed significant enrichment. Molecular function analysis further identified chromatin binding (GO:0003682; 261 genes; adjusted *p* = 6.01 × 10^−16^), transcription factor binding (GO:0008134; 261 genes; adjusted *p* = 1.87 × 10^−14^), transcription coregulator activity (GO:0003712; 227 genes; adjusted *p* = 3.16 × 10^−13^), DNA‐binding transcription activator activity (GO:0001216; 188 genes; adjusted *p* = 2.98 × 10^−8^), and DNA‐binding transcription repressor activity (GO:0001217; 137 genes; adjusted *p* = 1.66 × 10^−14^). Consistently, ATP‐dependent chromatin remodeling (hsa03082; 46 genes; adjusted *p* = 3.59 × 10^−4^) and Polycomb repressive complex (hsa03083; 45 genes; adjusted *p* = 1.33 × 10^−5^) were significantly enriched in the KEGG analysis.

The second functional theme comprised biological processes involved in protein homeostasis and the ubiquitin–proteasome system. Proteasome‐mediated ubiquitin‐dependent protein catabolic process (GO:0043161; 197 genes; adjusted *p* = 7.73 × 10^−6^) and positive regulation of catabolic process (GO:0009896; 218 genes; adjusted *p* = 5.68 × 10^−6^) were the principal enriched biological processes, whereas ubiquitin ligase complex (GO:0000151; 124 genes; adjusted *p* = 8.81 × 10^−3^) represented the major cellular component. The KEGG pathway Ubiquitin mediated proteolysis (hsa04120; 60 genes; adjusted *p* = 2.40 × 10^−3^) was also significantly enriched.

The third theme was associated with development, differentiation, and morphogenesis. Stem cell differentiation (GO:0048863; 121 genes; adjusted *p* = 3.75 × 10^−5^), epithelial cell development (GO:0002064; 100 genes; adjusted *p* = 7.91 × 10^−5^), epithelial cell proliferation (GO:0050673; 168 genes; adjusted *p* = 2.50 × 10^−4^), and focal adhesion (GO:0005925; 166 genes; adjusted *p* = 2.35 × 10^−4^) were significantly enriched. KEGG analysis further identified enrichment of the TGF‐beta signaling pathway (hsa04350; 51 genes; adjusted *p* = 3.59 × 10^−4^) and the Notch signaling pathway (hsa04330; 30 genes; adjusted *p* = 2.07 × 10^−3^).

The fourth theme represented stress response and cellular maintenance. Regulation of cellular response to stress (GO:0080135) was significantly enriched, involving 213 genes (adjusted *p* = 4.43 × 10^−6^). Two additional KEGG pathways, Autophagy—animal (hsa04140; 73 genes; adjusted *p* = 1.20 × 10^−3^) and Longevity regulating pathway (hsa04211; 44 genes; adjusted *p* = 9.16 × 10^−4^), were likewise significantly enriched. Together, these enrichment analyses demonstrate that shared exonic SSR‐containing genes are primarily associated with transcriptional regulation, protein turnover, developmental processes, and cellular stress‐response pathways across the three diving duck species.

To further explore whether species‐specific gene sets showed distinct functional tendencies, species‐specific exonic SSR‐containing genes were analyzed separately. Species‐specific enrichment analyses yielded limited and heterogeneous signals, with significant enrichment detected mainly in the *A. ferina‐*specific set, whereas no significant GO or KEGG enrichment was detected for *A. marila* and only two BP terms were significant for *N. rufina* (Supplementary Table S4). These results did not support a consistent functional pattern across the three species‐specific gene sets. Therefore, subsequent comparative interpretation focused on the shared exonic SSR‐containing gene set, while recognizing that species‐specific genes may contain lineage‐specific or species‐specific functional signals that were not fully resolved by the present annotation and enrichment framework.

### 3.4 Gene‐Architecture Bias in Exonic SSR‐Containing Genes

Because gene and exon length may influence the probability of detecting exonic SSRs, we further compared summed annotated exon length and gene genomic span between the shared exonic SSR‐containing genes and independent background genes in each species (Supplementary Table S5; Supplementary Figure S1). The independent background sets contained 8348 genes per species after symbol‐level deduplication and exclusion of genes represented in the shared foreground set. Shared exonic SSR‐containing genes had significantly longer summed annotated exon lengths than background genes in all three species. The median annotated exon lengths were 7664.5 bp versus 4622.5 bp in *A. ferina* (*p* = 5.83 × 10^−89^), 6338 bp versus 3304.5 bp in *A. marila* (*p* = 2.38 × 10^−176^), and 4529 bp versus 2159.5 bp in *N. rufina* (*p* < 2.2 × 10^−16^), corresponding to median ratios of 1.66, 1.92, and 2.10, respectively. Similarly, shared genes showed greater gene genomic spans than background genes, with median gene lengths of 23,500 bp versus 15,370.5 bp in *A. ferina* (*p* = 6.12 × 10^−53^), 23,085.5 bp versus 14,474.5 bp in *A. marila* (*p* = 1.85 × 10^−66^), and 22,005 bp versus 12,920 bp in *N. rufina* (*p* = 4.77 × 10^−83^). These results indicate that gene and exon architecture may contribute to the composition of exonic SSR‐containing gene sets and should be considered when interpreting their functional enrichment patterns.

## 4 Discussion

### 4.1 Cross‐Species Sharing of Exonic SSR‐Containing Genes

The most prominent comparative pattern emerging from this study is the contrast between the highly dynamic genome‐wide SSR landscape and the substantial cross‐species sharing of exonic SSR‐containing genes. Microsatellites are among the most rapidly evolving components of eukaryotic genomes, with variation arising through processes such as replication slippage and unequal recombination (Ellegren 2004; Gemayel et al. 2010). Consistent with this general pattern, the three diving duck species exhibited pronounced differences in genome‐wide SSR abundance and motif composition despite their similar genome sizes, GC contents, and assembly quality. In particular, the markedly reduced number of hexa‐nucleotide repeats in *N. rufina* indicates substantial interspecific variation in genome‐wide repeat composition within the sampled species. Comparable variation in genome‐wide microsatellite abundance has also been reported in other waterfowl, including mallard and Muscovy duck (Safaa et al. 2023).

In contrast, 4336 exonic SSR‐containing genes were shared among all three species after gene‐symbol standardization. This result indicates substantial sharing at the gene‐membership level. However, the present analysis did not compare orthologous SSR loci with respect to genomic position, repeat motif, repeat length, or repeat structure. Therefore, gene‐level sharing should not be interpreted as sequence‐level conservation of individual SSR loci. Previous studies have reported higher conservation of SSRs in certain functionally constrained genomic regions, including coding regions, compared with genome‐wide SSRs (Metzgar et al. 2000; Li et al. 2004; Gemayel et al. 2010). However, the present study did not distinguish coding exons from untranslated exons and therefore cannot determine whether such constraints explain the observed cross‐species sharing.

An additional line of evidence supporting this interpretation is the contrasting repeat composition between genome‐wide and exonic SSRs. Whereas mono‐ and hexa‐nucleotide repeats predominated at the genome level, exonic SSRs were consistently enriched for di‐nucleotide repeats across all three species. The shared gene‐level pattern may reflect the fact that many genes involved in fundamental cellular processes are represented across closely related species. However, the present data do not establish that exonic SSRs themselves are subject to stronger functional constraint, nor do they demonstrate that the SSRs directly contribute to the functions of the host genes. Instead, the results indicate that genes containing exonic SSRs are frequently shared across the sampled species and are enriched in several broad functional categories. These associations may reflect a combination of gene function, genome annotation, sequence composition, and gene‐architecture effects.

Collectively, these findings indicate that genome‐wide SSRs and exonic SSRs follow distinct evolutionary trajectories. Genome‐wide microsatellite landscapes appear to evolve primarily through lineage‐specific repeat expansion and contraction. Comparable lineage‐dependent variation in genome‐wide SSR abundance has also been documented in other vertebrate groups, including Euarchontoglires mammals and reptiles, indicating that rapid turnover of microsatellite landscapes is a widespread feature of vertebrate genome evolution (Song et al. 2021; Zhong et al. 2024). An additional difference between genome‐wide and exonic SSRs was observed in their repeat composition. Whereas mono‐ and hexa‐nucleotide repeats were prominent at the genome level, di‐nucleotide repeats predominated among exonic SSRs in all three species. This contrast indicates that SSR composition varies across genomic contexts and may reflect differences in local sequence characteristics and gene architecture. The shared gene‐level pattern further suggests that genes containing exonic SSRs are represented across the three sampled species. However, the present analysis cannot determine whether this sharing results from conservation of individual SSR loci, host‐gene characteristics, annotation consistency, or other evolutionary processes. Comparative analyses at the orthologous locus level, including repeat motif, repeat length, genomic position, and sequence context, will be required to distinguish among these possibilities.

### 4.2 Functional Associations With Transcriptional and Epigenetic Regulation

Functional enrichment analyses further revealed that the shared exonic SSR‐containing genes were not randomly distributed across the tested functional categories but showed significant enrichment in processes related to gene regulation. Among the four functional themes identified, gene transcription and epigenetic regulation represented the dominant enrichment signature, encompassing chromatin remodeling, transcription regulator complexes, transcription factor binding, epigenetic regulation of gene expression, RNA processing, alternative mRNA splicing, and Polycomb‐associated pathways. The consistency of these enrichments across Gene Ontology and KEGG analyses indicates that transcriptional and epigenetic regulation represents a major functional context among the shared exonic SSR‐containing genes.

Previous studies have demonstrated that variation in coding repeat length can influence protein conformation, transcriptional activity, RNA processing, and translational efficiency, thereby generating quantitative modulation of gene function without necessarily altering gene identity (King et al. 1997; Gemayel et al. 2010). Accordingly, the enrichment of exonic SSR‐containing genes within regulatory pathways indicates an association with genes in transcriptional and epigenetic regulation.

The enrichment of chromatin‐remodeling complexes and Polycomb‐related pathways is particularly noteworthy because these systems occupy central positions in vertebrate epigenetic regulation. ATP‐dependent chromatin remodelers regulate DNA accessibility by altering nucleosome organization, whereas Polycomb group proteins establish and maintain transcriptionally repressive chromatin states that are essential for developmental programming and cellular identity (Margueron and Reinberg 2011; Clapier et al. 2017; Schuettengruber et al. 2007). Likewise, the enrichment of alternative mRNA splicing highlights another important layer of post‐transcriptional regulation. Alternative splicing substantially expands transcriptomic and proteomic diversity and represents a major mechanism underlying developmental plasticity and environmental responsiveness in vertebrates (Nilsen and Graveley 2010; Baralle and Giudice 2017). The representation of exonic SSR‐containing genes in these regulatory pathways suggests that genes involved in transcriptional and epigenetic regulation constitute an important functional component of the shared gene set.

Importantly, these findings should not be interpreted as demonstrating that exonic SSRs directly regulate transcription or alternative splicing. Rather, they indicate that genes harboring exonic SSRs are significantly overrepresented within regulatory pathways. Distinguishing among these scenarios will require future studies integrating population genomics, comparative transcriptomics, and functional experiments.

Taken together, the enrichment of these regulatory pathways across the three sampled species indicates that genes containing exonic SSRs are associated with transcriptional and epigenetic regulatory functions in the present comparative dataset. Whether similar associations are widespread across Aythyini remains to be determined with broader taxonomic sampling.

### 4.3 Functional Associations With Protein Homeostasis and Development

Beyond transcriptional regulation, the shared exonic SSR‐containing genes were enriched in pathways associated with protein homeostasis, cellular differentiation, and tissue development. Although these functional categories encompass diverse biological processes, they converge on a common theme: the maintenance of cellular integrity throughout development and adult life. Their enrichment indicates that shared exonic SSR‐containing genes are associated with fundamental regulatory and cellular‐maintenance functions. Among these pathways, the ubiquitin–proteasome system (UPS) represents one of the most fundamental mechanisms responsible for maintaining proteome stability. The significant enrichment of ubiquitin‐mediated proteolysis, ubiquitin ligase complexes, and proteasome‐associated biological processes therefore indicates that shared exonic SSR‐containing genes are associated with core protein quality‐control functions. These pathways are consistent with the central role of protein quality control in maintaining cellular homeostasis (Ciechanover 2005; Dikic 2017).

Developmental categories included stem cell differentiation, epithelial development, epithelial proliferation, focal adhesion, and both TGF‐β and Notch signaling. These pathways are broadly involved in cell fate determination, tissue morphogenesis, and intercellular signaling (Artavanis‐Tsakonas et al. 1999; Massagué 2012). These results indicate that the shared exonic SSR‐containing genes are associated with protein homeostasis and developmental functions. They do not establish that the SSRs themselves are responsible for these enrichments. The observed associations may be influenced by gene function, sequence characteristics, annotation coverage, and gene architecture.

### 4.4 Stress‐Response Pathways in an Ecological Context

Among the four functional themes identified, stress response and cellular maintenance showed enrichment in regulation of cellular response to stress, autophagy, and the KEGG longevity‐regulating pathway. Autophagy is a conserved mechanism involved in the removal of damaged cellular components and maintenance of cellular homeostasis under conditions including nutrient limitation, oxidative stress, and hypoxia (Mizushima and Komatsu 2011; Levine and Kroemer 2019; Aman et al. 2021). These pathways therefore indicate that shared exonic SSR‐containing genes are associated with cellular processes involved in stress response and maintenance.

These findings can be considered in the physiological context of diving ducks, which experience repeated episodes of apnea and reoxygenation during underwater foraging. Such conditions can impose challenges related to oxygen availability and oxidative stress (Meir et al. 2009; Hochachka and Lutz 2001; Cole et al. 2022; Xia et al. 2026). However, the present study did not directly examine hypoxia‐responsive gene expression, physiological phenotypes, or the functional effects of individual SSRs. The observed enrichment should therefore be regarded as hypothesis‐generating rather than as evidence of a diving‐specific adaptive signature.

Broader taxonomic sampling, population genomic analyses, transcriptomic studies, and functional experiments will be required to determine whether these associations are widespread among diving ducks and whether particular exonic SSRs contribute to physiological responses relevant to diving.

### 4.5 Evolutionary Implications, Study Limitations and Future Perspectives

This study provides a comparative framework for examining genome‐wide and exonic SSR patterns in three diving duck species. Genome‐wide SSR abundance and repeat composition varied substantially among species, whereas exonic SSR abundance showed more similar values after normalization by total annotated exon sequence length. A total of 4336 exonic SSR‐containing genes were shared among the three species based on standardized gene symbols, and these genes were enriched in functional categories related to gene regulation, protein homeostasis, development, and stress and cellular maintenance. These results describe cross‐species sharing and functional associations among genes containing exonic SSRs, but do not establish sequence‐level conservation of individual SSR loci or direct functional effects of the SSRs themselves.

Several limitations should be considered. First, cross‐species gene comparison was based on standardized gene symbols rather than a dedicated one‐to‐one orthology‐inference pipeline. Functional annotation relied on human annotation resources because an Anatidae‐specific functional annotation database was unavailable. Although this approach enabled the use of standardized GO and KEGG resources, it may reduce functional resolution for avian‐specific or lineage‐specific genes and may introduce ambiguity associated with duplicated symbols and paralogous relationships. The enrichment results should therefore be interpreted as functional associations based on cross‐species identifier mapping rather than as evidence derived from formally established one‐to‐one orthologs. Second, the common background gene universe was constructed from the intersection of the three species‐specific background sets to improve comparability among species. This strategy reduced differences in annotation coverage but excluded genes represented in only one or two species. Consequently, the enrichment results reflect the shared analyzable gene space rather than the complete functional repertoire of each species. Species‐specific exonic SSR‐containing gene sets were also examined, but their enrichment signals were limited and heterogeneous. Their biological significance therefore remains to be evaluated with broader comparative sampling and more species‐specific annotation resources. Third, the analysis identified SSRs overlapping annotated exon features. Depending on the underlying annotation, exon features may include coding and untranslated exon regions. Accordingly, the present results should be interpreted as exonic SSR associations rather than as evidence restricted to protein‐coding sequences. In addition, the shared exonic SSR‐containing genes showed greater summed exon lengths and gene genomic spans than the corresponding background genes. Longer genomic regions provide more opportunities for SSR occurrence and detection, indicating that gene architecture may contribute to the composition of the foreground gene set. The present analysis assessed this potential source of bias but did not statistically remove it; future studies using length‐matched backgrounds or length‐aware models would help determine its contribution to the observed enrichment patterns.

Finally, only three diving duck species were included, and no non‐diving duck or broader outgroup was examined. The present findings therefore cannot be generalized to Aythyini, Anatidae, or diving birds as a whole. The stress‐response and autophagy‐related associations discussed above provide hypotheses for future investigation rather than evidence of a diving‐specific adaptive signature. Broader taxonomic sampling, orthology‐based locus comparisons, population genomic analyses, transcriptomics, and functional validation will be required to determine the evolutionary distribution and biological significance of exonic SSRs in waterfowl.

## 5 Conclusion

This study provides a comparative genomic characterization of genome‐wide and exonic simple sequence repeats (SSRs) in three diving duck species, *Aythya ferina* , *Aythya marila* , and *Netta rufina* . Genome‐wide SSR abundance and repeat composition varied substantially among species, whereas exonic SSR abundance showed more similar values after normalization by total annotated exon sequence length. A total of 4336 exonic SSR‐containing genes were shared among the three species based on standardized gene symbols, while each species also possessed a smaller set of species‐specific genes. Functional enrichment analysis of the shared gene set identified four broad functional themes involving transcriptional and epigenetic regulation, protein homeostasis, development and morphogenesis, and stress response and cellular maintenance. These findings indicate that genes containing exonic SSRs are associated with several fundamental cellular functions in the sampled species.

The present study assessed gene‐level sharing rather than conservation of orthologous SSR loci. The functional analyses also relied on cross‐species identifier mapping and a common background gene universe, while gene‐architecture analysis indicated that shared exonic SSR‐containing genes had greater summed annotated exon lengths and gene genomic spans than independent background genes. Therefore, the observed enrichment should be interpreted as an association of exonic SSR‐containing genes rather than as evidence for direct SSR‐mediated regulation, adaptive evolution, or sequence‐level SSR conservation.

The results provide a comparative genomic resource for future investigations of the evolution of exonic SSRs in waterfowl. Broader taxonomic sampling, orthology‐based locus comparisons, length‐aware enrichment approaches, and functional validation will help clarify the evolutionary distribution and biological significance of individual exonic SSRs.

## Author Contributions

**Jie Huang:** conceptualization (lead), methodology (lead), writing – original draft (lead), writing – review and editing (lead). **Bo Yang:** data curation (equal), methodology (equal). **Jiayi Qiao:** data curation (equal), methodology (equal). **Huaming Zhong:** data curation (equal), methodology (equal). **Chengzhong Yang:** funding acquisition (lead), project administration (lead), supervision (lead).

## Funding

This work was supported by the National Natural Science Foundation of China (Grant No. 31501845) and the Department of Science and Technology of Henan Province, China (Grant Nos. 222102320442 and 232102320299).

## Conflicts of Interest

The authors declare no conflicts of interest.

## Supporting information


**Supplementary Table S1:** Additional genome assembly and annotation information for the three diving duck species.

**Supplementary Table S2:** Gene Ontology enrichment results for shared exonic SSR‐containing genes.

**Supplementary Table S3:** KEGG pathway enrichment results for shared exonic SSR‐containing genes.

**Supplementary Table S4:** Species‐specific GO and KEGG enrichment results for the three diving duck species.

**Supplementary Table S5:** Gene and annotated exon length comparison between shared exonic SSR‐containing genes and background genes in three diving duck species.

**Supplementary Figure S1:** Comparison of summed annotated exon length and gene genomic span between shared exonic SSR‐containing genes and independent background genes in three diving duck species. Boxplots show the distributions of summed annotated exon length (A) and gene genomic span (B) for shared and background gene sets in *A. ferina* , *A. marila* , and *N. rufina* . Shared genes were compared with independent background genes after excluding genes represented in the shared foreground set. Summed annotated exon length was calculated by summing the lengths of exon features assigned to the same gene, whereas gene genomic span was defined as the genomic span of the annotated gene feature. Statistical significance was assessed using two‐sided Mann–Whitney U tests. The y‐axes are shown on logarithmic scales.

## Data Availability

The genome assemblies for *Aythya ferina* , *Aythya marila* , and *Netta rufina* analyzed in this study are publicly available from the NCBI GenBank database under accession numbers GCF_964211825.1, GCF_965140915.1, and GCF_964035555.1, respectively. Additional assembly information is provided in Supplementary Table S1. GO and KEGG enrichment results are provided in Supplementary Tables S2 and S3, respectively. Species‐specific enrichment results and gene‐architecture comparisons are provided in Supplementary Tables S4 and S5, respectively. Supplementary Figure S1 presents comparisons of annotated exon length and gene genomic span between shared exonic SSR‐containing genes and independent background genes. All data supporting the findings of this study are included in this article and its supplementary information files.
